# Transition from Acute Treatment to Survivorship: Exploring the Psychosocial Adjustments of Chinese Parents of Children with Cancer or Hematological Disorders

**DOI:** 10.3390/ijerph18157815

**Published:** 2021-07-23

**Authors:** Nelson Chun Yiu Yeung, Ka Chun Cheung, Ho Cheung Chau, Alex Wing Kwan Leung, Chi Kong Li, Teddy Tai Ning Lam, Ho Yu Cheng, Yin Ting Cheung

**Affiliations:** 1 Faculty of Medicine, JC School of Public Health and Primary Care, The Chinese University of Hong Kong, Hong Kong; 2 School of Pharmacy, Faculty of Medicine, The Chinese University of Hong Kong, Hong Kong; cedriccheungkc@gmail.com (K.C.C.); ivanchau31@gmail.com (H.C.C.); teddylam@cuhk.edu.hk (T.T.N.L.); 3 Department of Paediatrics & Adolescent Medicine, Hong Kong Children’s Hospital, Hong Kong; alexwkleung@cuhk.edu.hk (A.W.K.L.); ckli@cuhk.edu.hk (C.K.L.); 4 Department of Paediatrics, Faculty of Medicine, The Chinese University of Hong Kong, Hong Kong; 5 Hong Kong Hub of Paediatric Excellence, The Chinese University of Hong Kong, Hong Kong; 6 Little Life Warriors Society Hong Kong, Hong Kong; 7 Faculty of Medicine, Nethersole School of Nursing, The Chinese University of Hong Kong, Hong Kong; hycheng@cuhk.edu.hk

**Keywords:** caregiving, parents, childhood cancer, stress and coping, transition, survivorship

## Abstract

Objectives: Parents of children diagnosed with critical illnesses face multiple challenges during their caregiving experience. However, relevant studies have been limited in the Chinese context. Guided by the stress and coping model, we conducted a qualitative study to identify the stressors, coping strategies, and adjustment experiences of Hong Kong parents of children with cancer or hematological disorders. Methods: We recruited 15 parents of children with cancer or hematological disorders requiring bone marrow transplantation and were currently >2 years post-treatment. They participated in a 30-min semi-structured interview. Thematic analysis was performed using the grounded theory approach. Results: The stressors reported by parents included a high caregiving burden during their children’s diagnosis and treatment stages. The fear of recurrence, the need for information, and concerns about late effects were also common among the parents during their children’s transition/survivorship stage. To cope with these stressors, the parents commonly used problem-focused (e.g., seeking help from professionals and support groups) and emotion-focused (e.g., behavioral distractions, venting, and crying) strategies. Despite these stressors, parents reported positive changes through the caregiving experience, such as improved family relationships, developing health-protective habits, and the reprioritization of different aspects of life. Conclusions: Parents encounter different stressors during the cancer care continuum. Using different coping strategies, parents experience positive changes amidst caregiving. Future studies should explore culturally relevant adaptive coping strategies to enhance parents’ psychosocial adjustment.

## 1. Introduction

The survival rate of childhood cancers and serious chronic hematological disorders has increased significantly in the last two decades, yielding an emerging population of survivors in Hong Kong [1,2,3]. Unfortunately, survivorship comes at the cost of developing chronic health conditions due to intensive treatment modalities, including chemotherapy, radiotherapy, surgery, and hematopoietic stem cell transplantation (HSCT) [4,5]. It has been well documented in the literature that survivors may experience long-term physical effects and psychological distress, which may adversely affect their educational and occupational outcomes [5,6,7].

Care transitions are a major challenge for children and adolescents with cancer and hematological disorders [8,9]. In pediatric oncology/hematology, transition refers to the transfer of care from one provider and/or setting to another and also to the processes through which adolescents and young adults take on age-appropriate responsibility for their own health and healthcare [10,11,12,13]. When a child attempts to return to his/her normal life after cancer treatment, parents are often expected to take up key caregiving responsibilities [6,8]. Parents play a pivotal role in the care of a child with cancer; however, their psychosocial needs are often overlooked [11,14]. Although research has suggested that most parents are resilient and adapt well following the diagnosis, certain subgroups continue to report high levels of psychological distress throughout the care continuum [15,16].

Studies have suggested that adjusting to changes in the family environment and navigating the transition of care from the primary oncology team to the rehabilitation team may induce a psychological burden on the parents [6,15,17,18]. However, the majority of these findings are from Western countries and may not be applicable to the Chinese context due to cultural differences [19]. In one qualitative study in Hong Kong, nine Chinese parents of children with cancer were interviewed during the treatment stage [20]. The authors reported that Chinese cultural beliefs appeared to influence the parents’ coping experiences, with the parents tending to develop an attitude similar to the Chinese fatalistic belief of “yuan” (predetermined affinity) and to confront their child’s treatment with resigned acceptance [20]. In another recent study, greater parental resilience was associated with better quality of life in Chinese parents of children with cancer [16]. However, whether parents’ emotions and psychosocial challenges vary across different stages of cancer survivorship remains unknown.

In the process of cancer adaptation, parents also need to manage stress and solve specific caregiving-related challenges. The stress and coping model provides an appropriate framework for examining the important factors associated with psychosocial adjustments [21]. This model posits that people who encounter a potentially threatening event (e.g., diagnosis of a critical illness) usually go through primary and secondary appraisals and identify potential resources to cope with the associated stressors. The Stress and Coping model has been applied to understand adjustment among parents of children with chronic disease conditions [22,23]. For example, Beddard et al., found that different stressors (e.g., difficulties in obtaining an accurate diagnosis for their child, emotional changes and unmet supportive care needs) were common among parents of children with eye cancer [24]. Among Australian parents of children with autism spectrum disorder, multiple types of coping strategies (e.g., problem-focused, emotion-focused, social support seeking) were used to address caregiving-related stressors [25]. In addition, studies have also started to examine the positive changes experienced by parents. In the United Kingdom, one study showed that parents of children with an intellectual disability perceived some positive aspects from their caregiving experience, such as developing more meaningful relationships, changing life priorities and fostering greater appreciation of life [26]. Given that most of the studies examined parents’ experience in Western cultural contexts, whether the findings could apply to the adjustment among Chinese parents of childhood cancer survivors has yet to be explored.

The objective of this qualitative study is to describe the stressors and psychosocial adjustment experienced by Chinese parents of children with cancer or hematological disorders in Hong Kong during the transition and survivorship phases. Guided by the stress and coping model [21], we hypothesized that (1) the stressors experienced by parents differed throughout the cancer care continuum; (2) the parents used different types of coping strategies to deal with the caregiving-related stressors; and (3) despite the ongoing stressors, the parents experienced positive changes from their caregiving experience.

## 2. Methods

### 2.1. Study Design

A series of structured interviews were conducted between January and May 2020. Ethics approval was obtained from the Joint Chinese University of Hong Kong-New Territories East Cluster Clinical Research Ethics Committee. Written informed consent was obtained from all participants.

### 2.2. Subjects and Sampling

The participants were recruited from a local non-governmental organization (NGO) via a purposive snowball sampling approach. Parents were eligible for the study if they had a child who was diagnosed with cancer or an inherited hematological disorder requiring HSCT before the age of 18 years and were currently more than 2 years post-treatment. In addition, the parents were required to be able to speak Cantonese and read Chinese.

Research evaluating sample-size requirements in qualitative studies has shown that data saturation often occurs at approximately the 12th to 16th respondent in a homogenous group [27]. Therefore, the minimum target sample size for this study was 12 respondents. After the 12th participant, recruitment continued until data saturation was reached.

Fifteen parents (representing 10 survivors) were approached, and all agreed to participate in the study (response rate: 100%). Table 1 summarizes the participants’ demographic information and the children’s clinical characteristics. All 10 survivors represented in this study had undergone treatment for 4 to 12 years. Two thirds (*n* = 6) of the children were survivors of hematological disorders, whereas others (*n* = 4) were survivors of solid tumors. None of the survivors had any relapse or secondary malignancy. All the survivors resided in the city of Hong Kong and were previously treated in local public hospitals.

### 2.3. Study Procedure

Two investigators (HCC and KCC) conducted 10 semi-structured interviews. Couples (father and mother) were interviewed together. All interviews were conducted in Cantonese, an official spoken language in Hong Kong, and were audio-recorded upon consent. Each interview lasted between 30 and 40 min and was held either over the phone or via teleconference, due to coronavirus disease 19-related restrictions from February to April 2020.

Before the interview, the investigators briefly introduced the study objectives. An interviewer’s guide was designed to use an open-ended approach. The interviewer proceeded from the most general to the most specific questions, thus minimizing the influence of probing by the facilitators. The sample questions are presented in Table 2. The facilitators first asked the parents to narrate their perceived sources of stress throughout the cancer care continuum of diagnosis, active treatment, and survivorship. Subsequently, the general definitions of “transition period” and “survivorship” were introduced [8,9,28]. Specific details about stressors and coping strategies during these two periods were collected. Finally, the parents were asked to describe how their children’s experience had affected their family members, with a specific emphasis on their spouse and the children’s sibling(s) and grandparents.

Debriefings were conducted after every interview session to enhance the trustworthiness of the study. Two researchers (YTC and TTL) reviewed the interview sessions independently and identified deviations from the interview guide. The identified deviation was resolved through the meetings to maintain study fidelity. Dependability and confirmability were maintained by keeping an accurate record of the transcripts, retaining an audit trail, and resolving discrepancies through discussions among the research team members. Consistency was maintained through verbatim transcription and coding of the interview data. Transferability was maintained by providing comprehensive contextual information about the participants, study site and methodology for readers to establish accurate inferences of the qualitative results.

### 2.4. Data Analysis

All interviews were transcribed verbatim from the recording. Atlas.ti 8 (version 8, Berlin, Germany) was used to perform thematic analysis of the qualitative data. Two coders (HCC and KCC) first familiarized themselves with the transcripts and independently generated initial codes, which were then crosschecked. Discrepancies were resolved through consensus by two other independent investigators (LSY and YTC). This process was repeated until a final list of codes was generated. The codes were then collated into potential themes and reviewed by the research team to produce a description of the interview findings.

Grounded theory [29] was used to generate an explanation and identify the specific outcomes. Grounded theory [29] was chosen as the theoretical framework as it is one of the most widely used systematic approaches for analyzing qualitative data in the field of social sciences and healthcare [30,31]. Inductive reasoning was applied using the theories explained in the Introduction section. Thematic analysis was performed based on the principle of converting qualitative data into quantitative data [29]. The codes identified from the analyses were classified into the main coping strategies from the parents’ perspectives as outlined in the stress and coping model [21]. For reporting purposes, the themes, codes, and quotations were translated into English.

## 3. Results

Three major themes, namely sources of stressors, coping strategies, and psychosocial adjustment, were identified from the participants’ responses.

### 3.1. Theme 1: Sources of Stressors

#### 3.1.1. At Diagnosis and during Treatment

The parents recalled experiencing different stressors across the care continuum, which were broadly categorized as acute, chronic, and episodic stressors (Table 3). Acute stress was generated at the time of diagnosis, due to shock and increased psychological burden. Blame or misunderstanding from elderly family members contributed to episodic and chronic stress.

*As soon as I heard the diagnosis, it was as if time had stopped… Very shocking.*

—Parent #4, child with leukemia

*Of course, it was stressful at the time of diagnosis…immediately, I thought there’s no cure, he will die.*

—Parent #10, child with aplastic anemia

*The elderly in the family kept questioning why this misfortune happened to the child. We did not know the answer either.*

—Parent #12, child with leukemia

During treatment, the main stressors were from acute complications, the fear of death, and caring for the sick child at the hospital. Seeing negative outcomes in other patients also generated a large amount of stress. The parents also reported feeling emotionally stressed from having to deal with emergencies when their children fell sick at home.

*We made friends with the family in the neighboring bed in the ward, but we got upset when we saw his health deteriorate.*

—Parent #1, child with leukemia

*Staying at the ward was very tiring (as we need to take care of the child). We slept on a sofa bed... We felt drained physically and emotionally.*

—Parent #7, child with leukemia

*There was a time when our child developed some complications at home after returning from the hospital. It was too sudden and we were unprepared due to a lack of experience. We didn’t know what to do.*

—Parent #14, child with CNS tumor

#### 3.1.2. During the Transition and Survivorship Stages

After active treatment, most of the parents reported immediately seeking information from different sources to help their children transition back to normal life. Panic was greatest when there was insufficient information or conflicting information regarding the risk factors for relapse and late effects. Some of the parents also highlighted that searching for such information sometimes heightened their fear, rather than alleviating it.

*Stress was most intense when looking up information yourself. You don’t know what information to trust and what not to trust.*

—Parent #10, child with aplastic anemia

Although most of the parents did not show obvious signs of psychological distress during the interviews, all of them could articulate their existing concerns and fears regarding their children’s situation, even after the children had completed all curative treatments. The fear of relapse and the demands of caregiving generated substantial chronic stress for the parents. These stressors included negative experiences from a deteriorating health situation or news that the child of another family had relapsed.

*There are lots of things to think about, lots of planning…and a lot of precautions needed to prevent relapse and prevent side effects.*

—Parent #1, child with leukemia

*During transition, we are terrified of relapse…everything we do is to prevent relapse. Every time we collect the results of his MRI scans, I fear the worst…even right now, although it has been 6 years (since end of treatment) already.*

—Parent #3, child with soft-tissue sarcoma

*I served in an NGO, and I encountered families of children who relapsed. Whenever I attended their funerals, I wondered if my child would be one of them in the future.*

—Parent #4, child with leukemia

At the long-term survivorship stage, the fear of relapse persisted. Other chronic stresses came from academic or puberty concerns for their children. The prospect of losing constant monitoring from the pediatric clinic was concerning to some, as they did not have confidence that the “new” doctors in the adult clinics would understand their child’s complex medical history. This concern was apparent in one couple whose child was a survivor of CNS tumor.

*Now we are focused less on his health and more on his academics. It’s very tough for him, especially in Hong Kong. He is stressed, and we are stressed too. This is Hong Kong.*

—Parent #15, child with leukemia

*My son returned to school. It was great, but I needed to be around because he was in a wheelchair and I needed to help him use the restroom.*

—Parent #11, child with osteosarcoma

*My daughter needs to take medications for her entire life. She needs special attention in all aspects of her life. It seemed like after she turned 18 years old, she would lose all of the resources and support from the childhood cancer clinic.*

—Parent #13, child with CNS tumor

### 3.2. Theme 2: Coping Mechanisms

Based on the stress and coping model, we broadly categorized the coping strategies into problem-, appraisal-, and emotion-focused approaches (Table 4).

#### 3.2.1. Problem-Focused Coping

Researching out to and communicating with the members of support groups within the NGOs helped relieve stress by exchanging caretaking information. For example, the parents reported turning to other parents for information on the benefits and harms of using Chinese medicine during the survivorship period. Some of the parents actively sought help from social workers and teachers when their children returned to school.

*We don’t get any information about traditional Chinese medicine from the doctors. Sometimes, it’s the parents from the NGO and the social workers…they give more solid information and experiences (than nurses and doctors).*

—Parent #12, child with leukemia

*There are many other issues…you know, those non-health related ones…Doctors may not have time to deal with them. I have to actively ask other parents.*

—Parent #7, child with leukemia

*My son needed to use a wheelchair when he returned to school. Oh, I realized that the school is really good with offering special help…Sometimes, we tend to think too much…actually you just have to open your mouth and ask (for help).*

—Parent #11, child with osteosarcoma

A minority of the parents intended to seek professional help for their emotional distress, but they were admittedly very apprehensive about where they should go.

*The psychiatrist was very honest…He was only there for the child. If the parents need his services, they need to be on a separate waiting list. This didn’t make sense because it is actually a family problem!*

—Parent #13, child with CNS tumor

#### 3.2.2. Appraisal-Focused Coping

Many of the parents claimed that they had an optimistic outlook and were able to put a positive spin on their predicaments. Others selectively accepted positive medical prognoses as a method of coping.

*I have been good at handling stress ever since my son was diagnosed with cancer because I work in a fast-paced work environment. I can handle both work and family stress well.*

—Parent #8, child with leukemia

*We are very selective…we choose to believe only the positive comments made by the doctor, and ignore those comments that make us uncomfortable.*

—Parent #3, child with soft-tissue sarcoma

*We started to rely more on our (Christian) faith... If God decides to take her away, so be it. It took us a while though (to accept that).*

—Parent #2, child with leukemia

*We told ourselves—we need to win this war. Don’t show a bitter face all the time; that’s meaningless. Let’s do everything happily.*

—Parent #6, child with germ cell tumor

#### 3.2.3. Emotion-Focused Coping

Some of the parents used emotion-focused strategies to regulate their negative emotional reactions to the stressors. Most of the parents admitted to taking up sports, music, or meditation as a method of stress relief. One parent picked up a new skill as a method of distraction and self-enrichment. They also noted crying as a form of emotional relief.

*The PWH (Prince of Wales Hospital) is geographically close to CUHK (The Chinese University of Hong Kong), so I registered for some courses at CUHK after my child’s health stabilized. I think it (learning) is a big distraction, so I won’t be so focused on my child’s condition.*

—Parent #7, child with leukemia

*Caring for a sick child can be very “chur” (a colloquial Cantonese phrase for the term “demanding”). Sometimes I cry and let it out, or I would take a walk on my own… I do it alone. You need to have some time off from the stressful situation.”*

—Parent #13, child with CNS tumor

*I listened to music, downloaded a whole list of songs to make myself feel more relaxed.”*

—Parent #15, child with leukemia

### 3.3. Theme 3: Positive Aspects of Caregiving

#### 3.3.1. Improved Family Relationships and Dynamics

Many of the couples expressed that spousal relationships generally improved and siblings were more actively involved in caretaking. Occasionally, sibling jealousy occurred because of the parents’ focus on the sick child. One couple mentioned that the child’s grandparents were not informed about the grandchild’s disease until it had been resolved, while another couple chose not to inform them at all.

*We (my husband and I) became more focused on God as a family. We talk things out when we have differences—this is something positive that came out of this ordeal.*

—Parent #1, child with leukemia

*His (the sick child’s) relationship with his sister has improved. Before this, they ignored each other. When her brother was first diagnosed with cancer…she asked, ‘Why don’t they (parents) care about me?’…In the end she understood that if she can take care of herself, then we (parents) can devote more attention to her sick brother.*

—Parent #8, child with leukemia

#### 3.3.2. Heightened Awareness of Daily Health Habits

The parents of children with hematological cancers/disorders in particular adopted extra measures regarding daily household practices. Home cleanliness was more strongly enforced, together with dietary restrictions (e.g., avoiding carcinogens and unhealthy food) and sleep hygiene. There were certain restrictions on the child’s daily routines and traveling limitations due to health concerns, especially during the transition stage. To minimize patient exposure to threats of infections, one couple mentioned that they made a major decision to learn how to drive and purchased a car.

*After he was discharged from the hospital, we decided to buy a car, because we were worried that he may fall sick from taking public transport. It was a major change! I had to learn how to drive…I didn’t know how to drive before his cancer diagnosis.*

—Parent #1, child with leukemia

*We seldom dine out since then (after treatment). We always had our dinners at home to prevent infection).*

—Parent #8, child with leukemia

*He (the child) bleeds easily so we need to be aware of where we put sharp objects at home… His immunity is another concern; everything must be clean. We trained ourselves to get used to wearing masks at crowded places (even before the COVID pandemic).*

—Parent #10, child with aplastic anemia

#### 3.3.3. Reprioritization of Different Aspects of Life

The parents reported obvious changes in social relationships and priorities during the survivorship stage. Some parents narrated that they minimized social activities while their children were undergoing treatment. However, after treatment, they gradually reestablished relationships with their network of friends, although they admitted that their social circles had become smaller and more selective.

*Our social life has changed. We stopped going to gatherings. We only kept some of our friends. Our social circle has been reorganized.*

—Parent #1, child with leukemia

*We find meaning and joy in helping other parents, especially those with children who are newly diagnosed with cancer. We have been through it, so we can tell them how to calm down and deal with their problems.*

—Parent #11, child with osteosarcoma

## 4. Discussion

The common stressors among parents of children with cancer or hematological disorders in the transition and survivorship periods were explored. As expected, we found that Chinese parents of childhood cancer survivors experienced different stressors during the transition and survivorship phases. These acute and chronic stressors persisted, even after the completion of curative treatment. Overall, the parents reported engaging in problem-focused (e.g., seeking help from professionals and support groups), appraisal-focused (e.g., changing perspectives), and emotion-focused (e.g., behavioral distractions, venting, and crying) coping strategies. Despite the stressors, some of the parents did report positive changes to their family dynamics, health habits, and reprioritization of life aspects, suggesting that caregiving may not be an entirely negative experience for these parents.

Addressing informational needs may help alleviate parental stress during the transition and survivorship periods [32,33]. One common barrier was the lack of adequate resources and referral pathways as the child transitioned into survivorship and adulthood. Similarly, pediatric oncologists/hematologists in China have acknowledged parents’ limited awareness of potential late effects as a barrier to implementing survivorship programs [34]. To address this problem, Chinese versions of the Children’s Oncology Group educational materials on late effects and local resources promoting the safe use of complementary and alternative medicine in children are now readily available in Hong Kong [35,36,37]. As the study participants were recruited through an NGO, it is not surprising that the parents emphasized the important role that the local NGOs play in promoting health education. Our finding suggests that NGO-led educational talks and support groups at easily accessible sites may help bridge the identified knowledge gaps and empower parents as they take up a caregiving role. Similar studies should also be conducted in other settings, such as the local hospitals and outpatient clinics, to explore other support mechanisms for caregivers during the transition and survivorship phases.

The sources of parental stress during active treatment included anxiety, fear of treatment-related complications, and interruptions at night due to caregiving demands. However, the parents still struggled with the psychological ramifications and expressed the need for psychological support, even after the completion of treatment. In one longitudinal study, although most of the children with cancer showed relatively good psychological adjustment, many caregivers continued to show elevated symptoms of anxiety and post-traumatic stress over time [38]. As highlighted by one participant, the psychological services available in the local healthcare setting are mainly focused on the child, with substantially less focus on the family members. This is an inherent limitation of the current healthcare system in Hong Kong. The recent centralization of pediatric oncology/hematology services in the first pediatric hospital in Hong Kong (the Hong Kong Children’s Hospital) has provided an unprecedented opportunity to develop comprehensive care for children with critical illnesses. Like many other institutions in developed countries, future effort should be targeted at delivering family-centered care to address the needs of caregivers too, though this goal requires deliberate effort of multiple stakeholders and policy makers.

Regarding coping strategies, our findings were consistent with other studies conducted in parents of children with different medical conditions. For example, a Taiwanese showed that parenting stress activated the use of emotion-focused coping and problem-focused coping among caregivers of children with cancer [39]. Among parents of children with autism spectrum disorder, problem-focused strategies (e.g., researching to expand understanding of child’s disease), restructuring problems in a positive way, engagements in “me-time” activities (e.g., exercise), and disengaging from stressors (e.g., behavioral distractions and venting) were commonly also reported [25]. These findings imply that parents of children with chronic medical conditions do adopt similar types of coping strategies when dealing with caregiving-related stressors. This study provided some insights on the commonly used coping strategies among Chinese parents of children with cancer or hematological disorders. Future studies should quantitatively examine how coping strategies are associated with parent’s well-being through longitudinal assessments and develop interventions to promote adaptive coping strategies among these parents.

Despite the stressors, most parents identified positive changes to their family dynamics and reprioritization of life aspects. Such dimensions of positive changes were in line with theoretical conceptualizations of an individual’s posttraumatic growth in response to negative life events [40]. This observation is also consistent with the positive experiences of childhood cancer survivors and their families [41] and prior research on parents of children with other chronic medical conditions such as intellectual disability [26]. One of the unique dimensions found in our study is related to heightened awareness of daily health habits. The interviewed parents reported positive changes in daily household practices and awareness of home cleanliness. This finding might be particularly relevant to Chinese families during the transition from active treatment to survivorship. One study compared Caucasian and Chinese families’ caregiving to children with cancer in the United States; authors observed that the Chinese families focused a great deal on the proper diet for the child in contrast to Caucasian families who largely put nutrition as a lower priority [19]. Future research should also investigate how these dimensions of positive changes differ across the adjustment journey in parents.

## 5. Limitations

We acknowledge several limitations of this study. The Prevention and Control of Disease (Prohibition on Group Gathering) Regulation (Cap. 599) came into effect on March 29, 2020, to prohibit any group gathering of more than four persons in any public place in Hong Kong [42]. As face-to-face interviews or focus group discussions were not viable, the participants’ non-verbal expressions or interactions could not be considered. Furthermore, the snowball sampling approach might have led to selection bias, because the participants were likely to recommend individuals with similar characteristics to their own. For example, two family units (four participants) shared the same faith and location of worship. The education attainment of adult survivors (66% with college education) seemed to be higher than the proportion estimate in the age-matched general population (40% of individuals aged 35–49 years old with college education) [43]. Finally, only a modest sample size of 15 parents was recruited and other major types of childhood cancers (e.g., lymphoma) and hematological disorders (e.g., thalassemia major and hemophilia) were not represented. As this study was conducted through an NGO, we did not have access to detailed medical information of the survivors, though they seemed to be in a good health status according to their parents. Hence, our findings may not be generalizable to other cohorts, such as parents with children who suffer from cancer relapse, secondary malignancy, or serious long-term effects such as chronic graft-versus-host disease. It has been suggested that the saturation of themes in a small-scale study usually occurs within 12 interviews for groups with targeted inclusion criteria [44], and our thematic analysis showed recurring themes after analyzing eight interview scripts. However, we also acknowledge that the survivors represented in this study had different cancers and were diagnosed at a different age, hence resulting in a heterogeneous sample. Nevertheless, the current sample size of 15 participants was thus deemed sufficient to generate preliminary data to advise further research directions in this area.

## 6. Conclusions

We identify some of the multifaceted stressors that Chinese parents of children with cancer or hematological disorders in Hong Kong experience during the care continuum, with a focus on identifying psychosocial needs during the transitional and survivorship stages. Oncology practitioners should proactively encourage parents to acknowledge and share their personal difficulties when taking care of their children. It is important to address different aspects of the stressors that parents face during the transition period and the provision of care when the child attempts to return to normal life. Our findings also emphasize the need for the extended provision of psychological support for parents, even after their children have completed active treatment. Reducing the sources of stress may lead to better psychosocial adjustments for parents of children with life-threatening conditions.

## Figures and Tables

**Table 1:** Demographic characteristics of participants.

	Parents’ Characteristics				Child’s Characteristics			
Participant ID (#)	Age (Years)	Role	Highest Education	Number of Children in the Family (Birth Order of the Survivor)	Sex	Current Age (Years)	Diagnosis (Age at Diagnosis in Years)	Years since End of Treatment
**#1**	47	Mother	College	3 (2nd)	F	14	Leukemia (<1)	12.5
**#2**	51	Father	Above college					
**#3**	47	Mother	College	1	M	16	Soft-tissue sarcoma (9.5)	5.8
**#4**	50+	Mother	College	2 (1st)	M	20	Leukemia (12)	6.1
**#5**	44	Mother	High school	2 (2nd)	M	22	Germ cell (17)	4.2
**#6**	47	Father	High school					
**#7**	47	Mother	Above college	2 (1st)	M	11	Leukemia (3)	4.3
**#8**	50+	Father	Above college					
**#9**	36	Mother	College	2 (1st)	M	10	Aplastic anemia (3)	4.8
**#10**	48	Father	High school					
**#11**	57	Father	College	2 (2nd)	M	16	Osteosarcoma (6)	9.1
**#12**	49	Mother	High school	1	M	14	Leukemia (3)	8.3
**#13**	53	Mother	College	1	F	17	CNS tumor (10)	6.6
**#14**	53	Father	College					
**#15**	50	Mother	High school	2 (2nd)	F	11	Leukemia (3)	5.2

CNS: central nervous system, F: female, M: male.

**Table 2:** Samples of interview questions.

Questions	Probes
Throughout the cancer care continuum, can you recall when did you experience the highest level of stress?	At diagnosis Making treatment decisions During active treatment Immediately after completion of treatment Early survivorship Long-term survivorship At the present moment
What are the sources of stress During active treatment After completion of treatment?	Fear of death Acute toxicities Work commitment Family commitment Caregiving at hospital setting Cancer recurrence Late effects Returning to normal life Maintaining good health (“*yeung san*”) Child’s development
How did you handle those stressors? What did you do to help yourself feel better?	(Problem focused) Seeking information, asking for help, time management, dividing caregiving responsibilities (emotion focused) crying, letting out emotions, meditation, distraction with other activities, reading, listening to music, relaxation techniques (appraisal focused) altering goals and values, identifying humor, bringing a positive spin
How has caring for a child with cancer changed your life, in terms of family, work, and social life?	Relationship with spouse Relationship with the sick child Relationship with the sick child’s siblings Relationship with the sick child’s grandparents Work and employment Relationship with colleagues at work Friends and social circle Financial difficulties

**Table 3:** Codes representing sources of parental stressors throughout the cancer care continuum.

Time Frame	Type	Source of Stressors	Frequency of Codes
Diagnosis	Acute	Initial cancer diagnosis	9
		Fear of death	6
		Questions from family members	4
		Worried that cancer is incurable	4
		Concerned about treatment duration	3
	Chronic	Constant blame from elderly in the family	2
		Juggling with child and elderly care	2
Active Treatment	Acute	Acute complications of treatment	10
	Chronic	Busy caring for child	9
		Physically demanding to care for child	8
		Concerned about late effects	4
		Seeing other patients struggle with complications	3
		Financial hardship	1
Transition	Chronic	Worried about relapse	8
		Not enough information on what to expect next	6
		Concerned about child’s physical and mental growth	5
	Episodic	Waiting for clinical reports during follow-up	3
Survivorship	Acute	Child fell sick all of a sudden	2
	Chronic	Worried about relapse	7
		Follow up visits to the doctor	6
		Lack of information on survivorship issues	6
		Concern about child’s academics performance	3
		Dealing with child’s puberty issues	3
		Waiting for clinical reports during follow-up	3
		Financial hardship	3
		Talking to siblings about cancer	1
		Explaining to child about his/her health status	1

**Table 4:** Codes representing parents’ coping mechanisms.

Mechanism (from Parents’ Perspectives)	Coping Strategies	Frequency of Codes
Problem-focused	Seek information to alleviate their worry	16
	Talk to other parents	10
	Talk to medical professionals	8
	Seek social support from NGOs	5
Appraisal-focused	Positive mentality	13
	Find meaning through faith	10
	Listen to advice selectively	7
	Find meaning through non-religious means	5
	Make peace with the reality of death	3
	Acknowledge that stress cannot be avoided	2
Emotion-focused	Exercise	6
	Crying	5
	Music	3
	Distraction by engaging in new hobbies/ learning new skills	3
	Meditation	2
	Work	2
	Stress eating	1

## Data Availability

The data presented in this study are available on request from the corresponding author. The data are not publicly available due to patient privacy concerns.
